# Crafting Nanostructured Hybrid Block Copolymer–Gold Nanoparticles by Confined Self-Assembly in Evaporative Droplets

**DOI:** 10.1021/acsmacrolett.4c00519

**Published:** 2024-09-23

**Authors:** Andrea Escher, Gianluca Bravetti, Simone Bertucci, Davide Comoretto, Christoph Weder, Ullrich Steiner, Paola Lova, Andrea Dodero

**Affiliations:** † Department of Chemistry and Industrial Chemistry, University of Genoa, Via Dodecaneso 31, 16146, Genoa, Italy; ‡ Adolphe Merkle Institute, University of Fribourg, Chemin des Verdiers 4, 1700, Fribourg, Switzerland; § Photonic Nanomaterials, Istituto Italiano di Tecnologia, Via Morego, 30, 16163 Genoa, Italy; ∥ National Center of Competence in Research Bio-Inspired Materials, Chemin des Verdiers 4, 1700, Fribourg, Switzerland

## Abstract

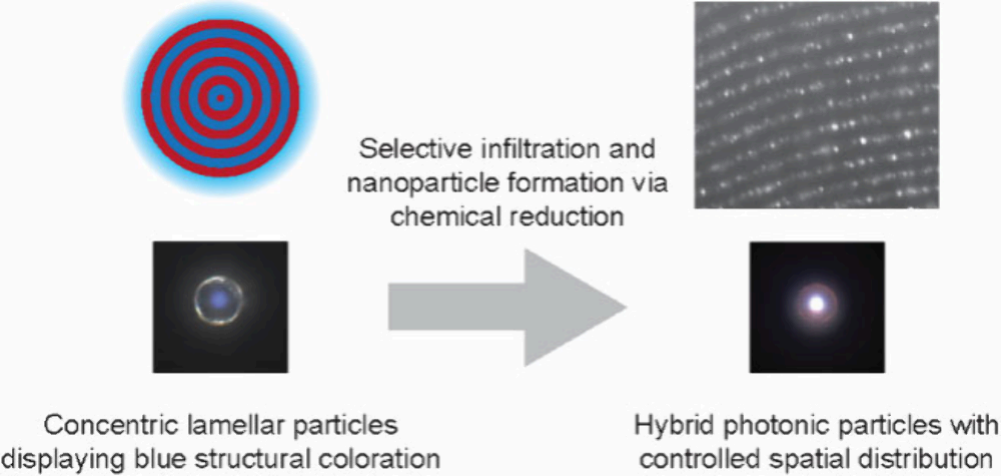

Hybrid organic–inorganic nanostructures offer significant potential for developing advanced functional materials with numerous technological applications. However, the fabrication process is often tedious and time-consuming. This study presents a facile method for fabricating block copolymer-based photonic microspheres incorporating plasmonic gold nanoparticles. Specifically, the confined self-assembly of poly(styrene)-*b*-poly(2-vinylpyridine) in emulsion droplets allows the formation of spherical, noniridescent, concentric lamellar structures, i.e., onion-like particles that are subsequently infiltrated with gold salt. Using ethanol as a preferential solvent allows the loading of metal ions exclusively into the poly(2-vinylpyridine) domains, which are subsequently reduced, leading to the *in situ*, spatially controlled formation of gold nanoparticles. The hybrid structures exhibit a well-defined photonic bandgap and plasmonic resonance at low gold concentrations. These results demonstrate the feasibility of fabricating optically active photonic structures comprising metal nanoparticles in a block copolymer array via a simple two-step fabrication process.

The relentless pace of technological advancement necessitates developing novel materials with advanced functionalities that are typically challenging to achieve through single-component systems, i.e., pure organic or inorganic materials.^1−3^ Polymer-inorganic hybrid materials present a promising avenue for exploration, offering unique opportunities to combine the advantageous properties of both components, making them prospective candidates for a diverse range of applications.^4−8^ Block copolymer-inorganic nanoparticle hybrids represent an emerging subset of this class of materials, exhibiting well-defined nanostructured morphologies that are useful for technological exploitation in photonics and related fields.^9−12^

Block copolymers (BCPs) comprise at least two distinct, covalently bonded macromolecular chains that are typically thermodynamically incompatible.^13^ They are well-documented to microphase separate as a function of their composition (i.e., the block volume fraction and interaction parameters between the blocks), resulting in a diverse range of complex hierarchical morphologies, including spheres, cylinders, bicontinuous gyroids, lamellae, and others.^14^ The domain periodicity of block copolymers can be controlled by exploiting different strategies.^14−17^ This ability has led to a rapid increase in the importance of block copolymers in fabricating advanced materials for a wide range of applications. In particular, self-assembled block copolymer morphologies are employed to fabricate photonic crystals (PhC).

PhCs are particularly interesting due to their ability to control light-matter interactions, which have been harnessed in several fields, including lasing and sensing.^18−21^ PhCs comprise periodic arrangements of at least two dielectric materials with disparate refractive indices, wherein the lattice parameters are commensurate with the optical wavelength of UV–vis or near-infrared light. Such lattices permit the formation of forbidden frequency regions for light propagation, namely photonic band gaps (PBGs), which can be identified by strong reflectance and impart the structure with distinctive structural coloration.

The three-dimensional confined self-assembly of block copolymers in emulsion droplets is an effective method for manufacturing photonic microparticles exhibiting a wide range of sizes, shapes, internal structures, and optical characteristics.^22−24^ These have also been integrated with an array of inorganic nanomaterials, including metal and metal oxide nanoparticles.^25−28^ However, the attained domain periodicities have not yet enabled the fabrication of PBGs in the visible spectral range, primarily due to the necessity for block copolymers with exceptionally high molecular weights.^9^

The present study aims to exploit a previously optimized strategy for manufacturing microsized block copolymer photonic particles exhibiting tunable PBGs and brilliant structural coloration.^29^ These outcomes are achieved through the confined self-assembly of linear diblock copolymers within emulsion droplets, which generates well-ordered concentric lamellar morphologies. The domain periodicity and the resulting displayed color are controlled by exploiting a supramolecular approach based on specific block-additive interactions. Here, such microparticles are subjected to the selective infiltration of a gold precursor (i.e., gold(III) chloride trihydrate) by swelling only one of the two lamellar domains in a water/ethanol mixture, exploiting univocal polymer–solvent interactions.^30^ Subsequently, a mild chemical reduction treatment with a sodium borohydride (NaBH_4_) ethanol solution at a controlled temperature enables the *in situ* growth of gold nanoparticles (AuNPs).^31^ This approach allows for the straightforward manipulation of the inorganic NP spatial location through selective infiltration of the block copolymer nanostructure, resulting in the periodic alternation of all-polymer and polymer-AuNP concentric layers. The complete fabrication procedure is depicted in Figure 1.

**Figure 1:**
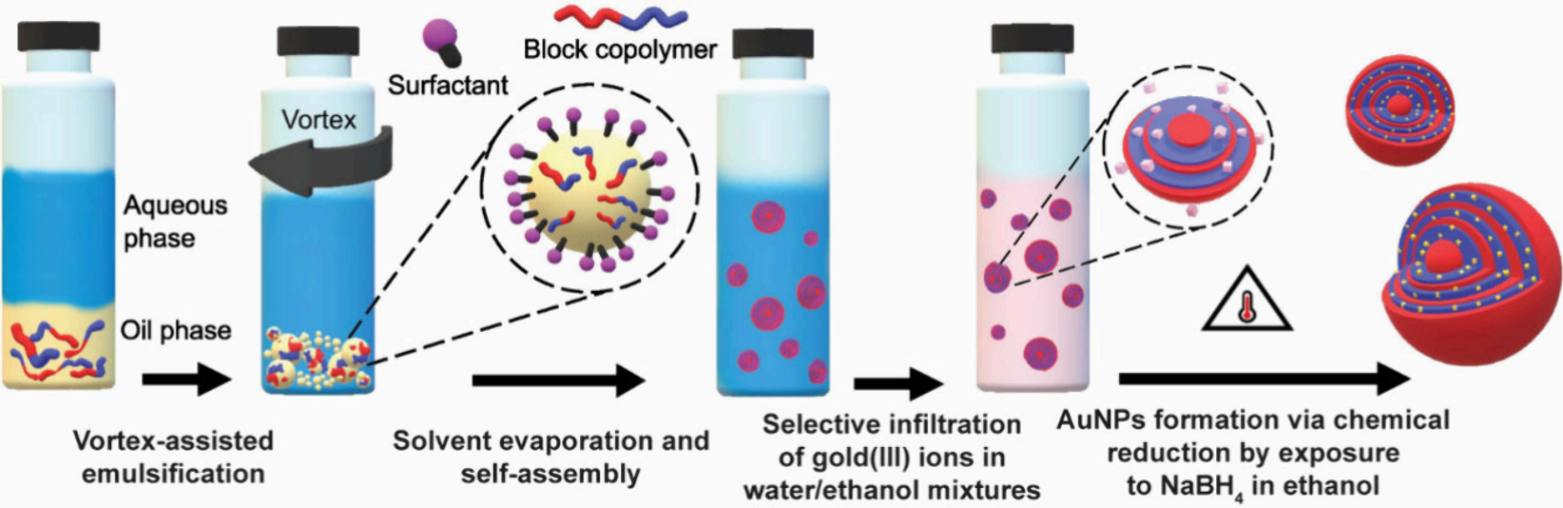
Schematic illustration of the preparation of hybrid microparticles consisting of BCPs and Au NPs. Concentric lamellar particles are first prepared by confined self-assembly of poly(styrene)-*b*-poly(2-vinylpyridine) in emulsion droplets (i.e., oil-in-water emulsion) using poly(vinyl alcohol) surfactant. The BCP particles are then infiltrated with gold(III) chloride in water/ethanol mixtures. The affinity of P2VP for ethanol ensures that only these domains are infiltrated with the metal precursor, while the amount of gold ions in the swollen structure is controlled by adjusting the water-to-ethanol volume ratio. Finally, AuNPs are synthesized *in situ* by exposing the infiltrated BCP particles to a NaBH_4_–ethanol solution at 50 °C overnight. This approach allows precise control over the spatial distribution of the NPs within the block copolymer architecture, forming well-ordered hybrid photonic structures.

To demonstrate the efficacy and simplicity of this methodology, poly(styrene)-*b*-poly(2-vinylpyridine) (PS-P2VP) particles are initially fabricated by confining the block copolymer self-assembly in emulsion droplets (an oil-in-water emulsion, where chloroform is used as the oil and poly(vinyl alcohol) is used as the surfactant to stabilize the emulsion). The resulting particles, whose size ranges between 5 and 20 μm, exhibit a reflection peak at approximately 435 nm, indicative of a blue color (as observed in the reflectance spectrum and optical microscopy image, Figure 2a and b, respectively). This phenomenon can be attributed to a well-defined concentric lamellar structure (Figure S1). Subsequently, the particles are infiltrated with gold(III) chloride, which has been dissolved at a concentration of 1 mM in water/ethanol mixtures with varying volume ratios. While the polar P2VP blocks exhibit good solubility in alcohol, the apolar PS blocks are insoluble in this solvent, limiting the accumulation of the ionic metal precursor to the P2VP domains.^32^ To elucidate the impact of the infiltrating medium in this process, the volume ratio of ethanol in the precursor solution is varied between 0 and 50%.

**Figure 2:**
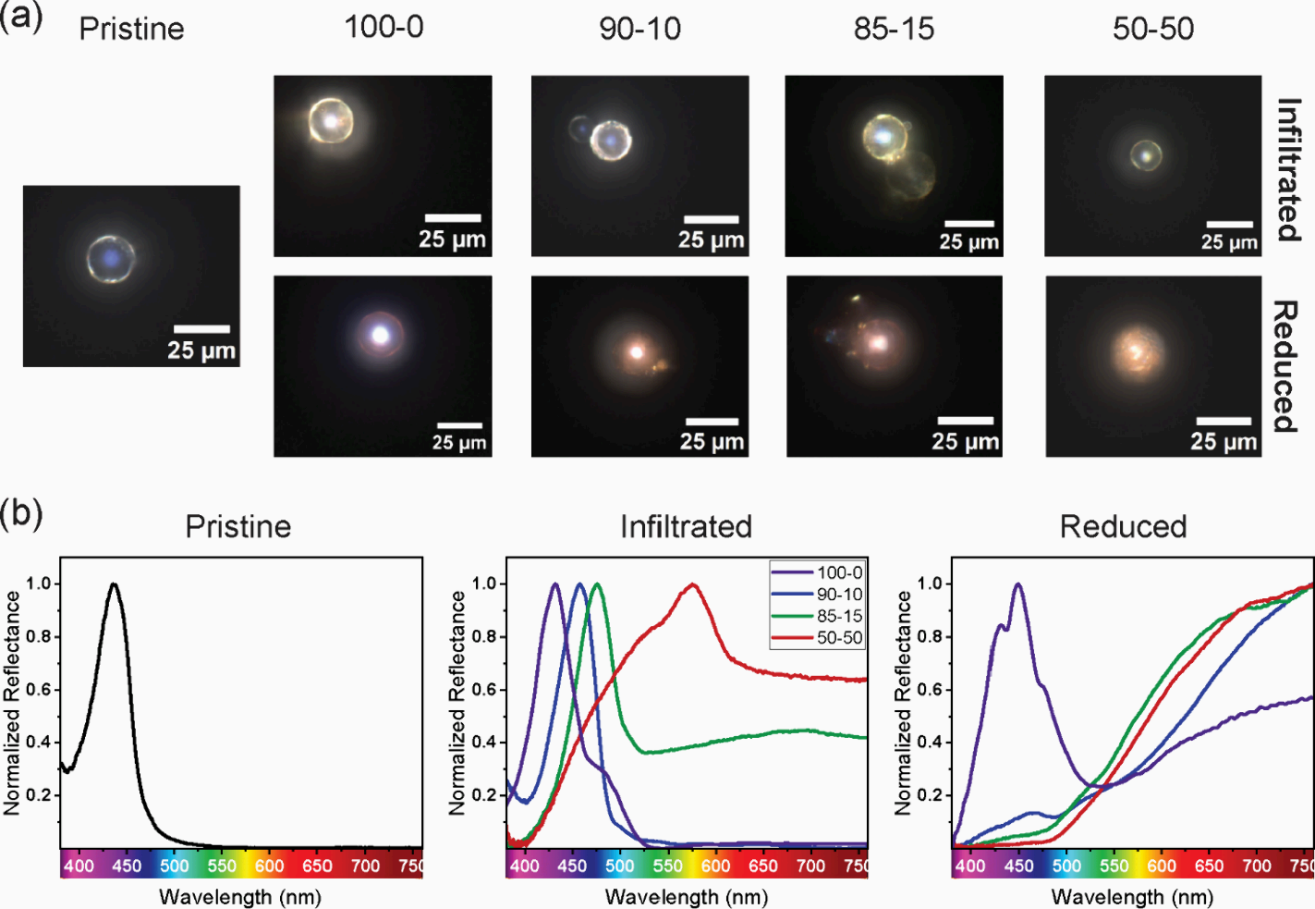
(a) Optical microscopy images and (b) normalized reflectance spectra of pristine BCP particles and hybrid particles before and after gold nanoparticle formation. BCP particles are infiltrated with a gold(III) solution from water/ethanol mixtures with water/ethanol volume ratios indicated in the top row. While the pristine particles show a clear blue color, the infiltration of the photonic particles with gold ions results in a yellowish hue. The structural color of the particles is preserved and red-shifted. After the formation of AuNPs by chemical reduction of gold(III) ions by exposing the infiltrated BCP particles to a NaBH_4_–ethanol solution at *T* = 50 °C overnight, the AuNPs take on a red color, replacing the optical signal of the photonic bandgap.

A comparison of the microscopy images presented in Figure 2a (pristine and infiltrated particles) reveals a notable change in the optical appearance of the photonic particles. Indeed, due to the gold ion infiltration, these particles exhibit a yellowish hue, primarily localized to the outer shell. Nevertheless, the photonic structure retains its distinctive structural coloration, which remains localized to the particle center. The reflectance spectra displayed in Figure 2b (infiltrated) provide further insights into the optical properties of the particles. While no significant changes can be discerned in the position and width of the PBG spectral peak for particles infiltrated with a gold solution in pure water (100–0), a gradual red shift of the reflectance peak is observed with increasing ethanol concentration. Notice that the strong reflectance of the 100–0 particle partially saturates the digital camera and, therefore, it appears white in the center with a blue-violet hue in the crown around the central area. Notably, the red shift of the PBG can be attributed to two distinct yet concurrent phenomena. First, an increase in ethanol concentration results in increased swelling of the P2VP layers, thereby increasing the domain periodicity.^33^ Indeed, it is reasonable to infer that the expansion of the P2VP layers exhibits only partial reversibility upon ethanol removal, resulting in thicker layers than their original size. This is because P2VP chains may undergo some conformational changes or reorganization during swelling due to the presence of ethanol. These changes might include stretching the polymer chains or reconfiguring physical cross-links between chains. Once ethanol is removed, the chains may not return to their original state due to the formation of new, stronger interactions or physical entanglements during the swelling process. Additionally, some ethanol molecules might remain trapped within the polymer matrix even after the bulk of the ethanol has been removed. This residual ethanol can continue interacting with the pyridine groups, preventing the polymer from fully returning to its original, unexpanded state. Furthermore, the rapid swelling in ethanol diminishes the overall organization of the lamellar structure, thereby widening the spectral width of the particle photonic bandgap and consequently lowering the color purity.^34,35^ The second effect is associated with the fact that upon increasing the ethanol volume in the infiltration mixture, more gold ions diffuse into the lamellar structure, which results in a progressive increase of the refractive index of the P2VP layers.^36−38^ Upon infiltration with the gold solution containing 15% ethanol, an additional broad peak between 500 and 750 nm is observed. This phenomenon can be attributed to the partial and inhomogeneous swelling of the external layers, which gives rise to the formation of a second photonic structure with a higher periodicity than the main one, corresponding to the inner layers of the particles. This phenomenon has been investigated in the literature,^39,40^ with studies reporting even more pronounced effects at an ethanol content of 50%. In this instance, the particles assume a green-yellow hue, and the corresponding reflectance peak broadens inhomogeneously. This effect is once more attributed to uncontrolled swelling of the P2VP layers and has been previously observed in the literature for planar structures infiltrated with solvents.^41^ The partial infiltration of the polymer layers is also corroborated by the electron microscopy micrographs shown in Figure 3a, which depict cross sections of the microspheres. As observed across the particle cross-section, the external layers exhibit a brighter appearance than the inner ones, indicating the presence of gold ions. As the ethanol content increases, the brightness of the internal layers rises, indicating that the ions can diffuse for a greater distance across the particle.

**Figure 3:**
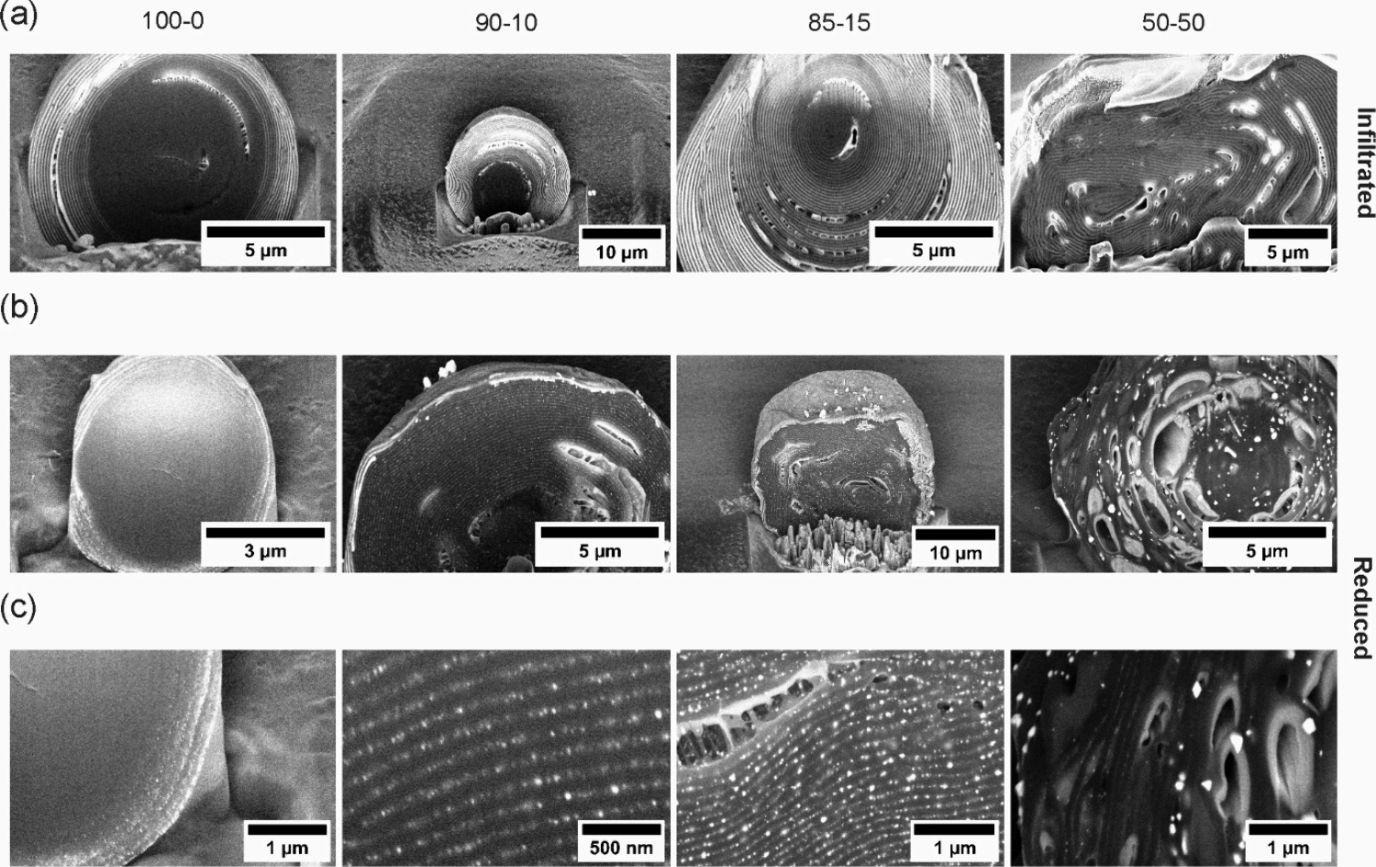
(a) FIB-SEM cross sections of PS-P2VP particles showing a concentric lamellar structure after selective infiltration of P2VP layers with gold ions in different swelling media. Increasing the volume ratio of ethanol leads to a more efficient diffusion of the metal precursor within the lamellae, as evidenced by a more pronounced contrast between the layers and the complete staining of the block copolymer structure. (b, c) Gold nanoparticles are synthesized *in situ* within the P2VP layers by reducing gold(III) ions by exposing the infiltrated BCP particles to a NaBH_4_–ethanol solution. The best results, i.e., homogeneous distribution of AuNPs and preservation of the concentric lamellar structure, are obtained at intermediate ethanol volume ratios (i.e., 90–10 and 85–15), where a homogeneous and complete distribution of nanoparticles within the lamellar structure is observed.

The formation of the gold nanoparticles via chemical reduction of the Au(III) ions with NaBH_4_ is then investigated. Figure 2a (reduced) demonstrates that only the particles infiltrated with gold ions in water (100–0) persist in exhibiting a discernible blue structural coloration with a reddish hue on the particle surface. As the ethanol concentration in the infiltration solution increases, the hue extends throughout the entire particle, indicating the presence of AuNPs with their characteristic plasmonic resonance.^42^ This behavior is reflected in the spectra of Figure 2b (reduced), where the relative intensity of the PBG at around 440 nm decreases significantly when the ethanol content is increased, with a second signal detected at longer wavelengths in all samples being associated with the plasmon resonance of the AuNPs. The successful formation of the nanoparticles is corroborated by the scanning electron microscopy micrographs shown in Figure 3b,c. The micrographs unambiguously demonstrate the presence of AuNPs, which are visible as bright spots within the microspheres due to their high electron density. However, an increase in the ethanol content results in a discernible enhancement of disorder in the photonic structure, inhibiting the necessary light interference for generating the PBG.

To gain further insight into the infiltration process and nanoparticle formation, focused ion beam scanning electron microscopy (FIB-SEM) is conducted on the pristine particles, particles infiltrated with the gold solution, and particles subjected to the chemical reduction reaction. Figure S1 depicts cross-section micrographs of the untreated particles following iodine staining. A distinct concentric lamellar structure devoid of imperfections is evident, with the PS layers appearing dark and the P2VP ones appearing bright.^43^ The structural analysis reveals a 65 ± 7 nm thickness for the PS and 63 ± 6 nm for the P2VP layers. Given that *n*_PS_ = 1.59 and *n*_P2VP_ = 1.62,^29^ the Bragg-Snell law at normal incidence yields λ_PBG_ = 410 nm, with *n* denoting the material refractive index and λ_PBG_ representing the bandgap spectral position. This prediction agrees with the experimental spectroscopy results presented in Figure 2b (pristine). Indeed, the slight discrepancy between the calculated PBG value and the experimentally measured value can be explained by conditions during the FIB-SEM analysis, in which the high vacuum and the high-energy ion beam cause shrinkage of the block copolymer domains and the consequent blue-shift of the reflected light.

In light of the selectivity of ethanol toward swelling the P2VP domains, a meticulous assessment is conducted to evaluate the impact of infiltration in diverse water/ethanol mixtures on the block copolymer structure. The corresponding FIB micrographs are presented in Figure 3a, wherein the layers containing gold ions (in the P2VP phase) appear bright. While the concentric lamellar structure is maintained in all samples, a discernible difference in the degree of infiltration efficiency is observed among them. Indeed, while infiltration of only a few external layers is observed when pure water is used as the infiltration medium, the entire lamellar structure becomes affected when ethanol is added. However, increasing the ethanol content above 15% causes defects in the block copolymer domains, including layer inhomogeneity and cracking, and loss of the lamellar structure is observed. This phenomenon can be attributed to the rapid and uncontrolled swelling of the P2VP domains, as evidenced by the spectra presented in Figure 2. The effect of ethanol content in the infiltration solution is assessed by evaluating changes in the domain periodicity, as reported in Figure S2a. As expected, independent of the ethanol content in the infiltration solution, PS layers do not present any significant thickness variations. In contrast, P2VP layers show a progressive thickness increment, which becomes relevant at a 50% ethanol concentration (i.e., *d*_P2VP_ = 123 ± 11 nm).

The formation of AuNPs via the chemical reduction of gold(III) ions by treating the infiltrated BCP particles with a NaBH_4_ ethanol solution (5 mM) can be readily discerned in all instances through the cross-section micrographs shown in Figure 3b and c. In this scenario, AuNPs are visible as bright spots scattered and dispersed exclusively in the P2VP domains. As anticipated, the dispersion aligns with the outcomes of the infiltration procedure, demonstrating optimal results when a moderate quantity of ethanol is employed. Under such conditions, the nanoparticles display a uniform distribution throughout the photonic structure and within each layer. In contrast, excessive ethanol not only disrupts the lamellar organization but also results in the aggregation of nanoparticles into large gold clusters, presumably due to precursor concentration gradients. No efforts are made to control the nanoparticle size distribution, which is likely a difficult undertaking since the internal layers of the particles contain a lower amount of gold ions and are likewise exposed to a different reducing agent concentration than the external ones. Additionally, it is worth mentioning that the solid polymeric layers likewise act as a physical barrier preventing the nanoparticle from leaching.

The influence of the nanoparticle synthesis on the lamellar structure is again evaluated by measuring changes in the layer thickness (Figure S3b). No significant variations can be detected compared to the infiltrated particles. Notably, in the case of the sample swelled in the 50–50 water/ethanol mixture, the disruption of the lamellar structure makes it impossible to perform any reliable analysis. Finally, to confirm that the disruption of the lamellar structure is associated with the solubility of P2VP layers in ethanol and is not, or only minimally, influenced by the nanoparticle formation, pristine BCP particles are swelled in a 50–50 water/ethanol mixture not containing gold ions. As clearly shown in Figure S3, the structure presents many defects. This finding indicates that the swelling and partial dissolution of P2VP domains upon ethanol exposure is the main reason for the deformation of the lamellar structure.

Further details concerning the fabricated hybrid photonic particles can be found in the small-angle X-ray scattering (SAXS) and ultrasmall-angle X-ray scattering (USAXS) data in Figure 4a. A distinctive scattering peak is evident in the case of Au(III) chloride-infiltrated particles (blue lines), which is attributed to the lamellar domains. The corresponding scattering vector *q* indicates a periodicity *D* of 143 nm for the particles infiltrated from water. A slight shift of the peak to smaller *q* values is observed for the samples treated in water/ethanol mixtures, as summarized in Table S1. This finding is consistent with the observation that the P2VP layers exhibit increased swelling with increasing the ethanol content in the infiltration solution.

**Figure 4:**
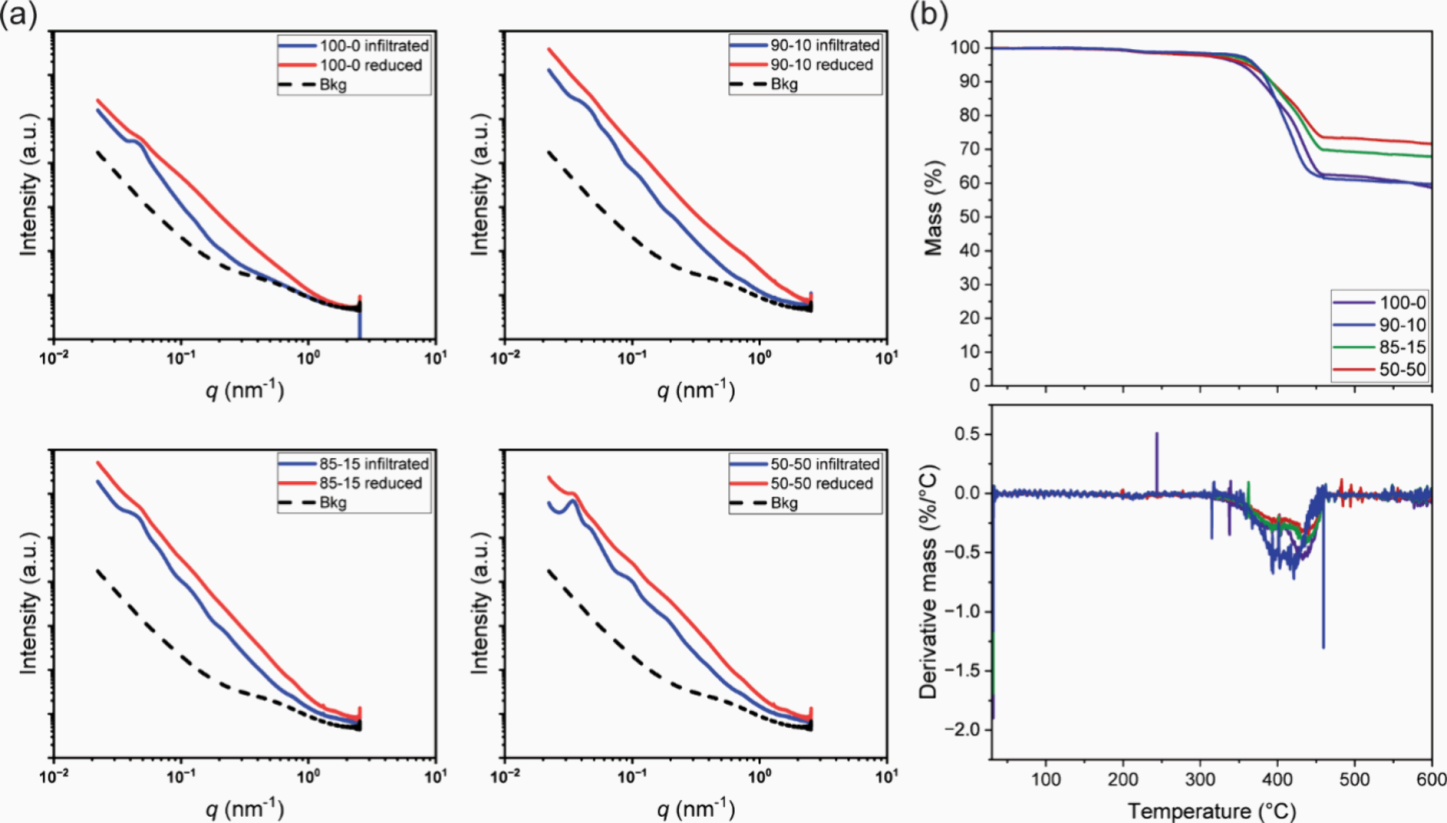
(a) Scattering spectra obtained from the combination of SAXS and USAXS measurements for block copolymer particles infiltrated (blue lines) in different swelling media and subsequently treated with NaBH to induce the formation of AuNPs (red lines). For the infiltrated particles, a distinct first-order scattering peak with a periodicity of about 150 nm is observed, which slightly increases with increasing ethanol volume ratio. The formation of the nanoparticles gave comparable results, although the scattering peak showed a lower contrast. (b) Thermogravimetric profiles and their derivatives are obtained in an air atmosphere with a heating rate of 10 °C/min, and a single degradation process is observed at about 400 °C. Increasing the ethanol volume ratio in the infiltration medium resulted in a higher inorganic residue at 600 °C, indicating a greater amount of AuNPs within the block copolymer structure.

Interestingly, after the nanoparticle formation through reduction, the intensity of the scattering peak (red lines) related to the lamellar structure shows a notable decline compared to that observed for the Au-salt infiltrated particles despite no discernible changes in the scattering vectors. This outcome can be explained by considering that as the content of AuNPs increases, the signal associated with the inorganic component dominates the overall scattering compared to the neat block copolymer domains.^44^

To further substantiate this explanation, the quantity of gold within the photonic particles is assessed through thermogravimetric analysis, with the corresponding data presented in Figure 4b. Despite the fact that it is not readily possible to determine the absolute amount of gold NPs embedded in the photonic structures, TGA traces show a clear dependence upon the ethanol content in the infiltration solution. In particular, while no significant changes are observed in the degradation profile, with the hybrid particles exhibiting stability up to a temperature of 350 °C, the residual mass at 600 °C can be attributed to the different content of AuNPs. Finally, the inorganic material constitutes approximately 60% of the total mass of the water-infiltrated particles. This value increases above 70% for the particles infiltrated in the mixture containing the highest ethanol content (Table S2), which again indicates a more effective infiltration of the gold ions due to the greater swelling of P2VP layers.

In conclusion, we demonstrate a straightforward and effective strategy for fabricating hybrid photonic microparticles comprising block copolymer concentric lamellar structures and gold nanoparticles. These composite structures can display a vibrant structural coloration associated with the periodic arrangement of the block copolymer domains and a plasmonic resonance effect characteristic of metal nanoparticles. The *in situ* growth of AuNPs by exploiting selective block solubility offers several advantages over other approaches commonly used in fabricating similar hybrid block copolymer structures. First, it ensures direct and precise control over the nanoparticle distribution of AuNPs within the polymeric phase. Second, the loading amount of the nanoparticles can be adjusted by either tuning the precursor concentration in the infiltration medium, which is not investigated in the present work, or by changing the solvent ratio to alter the swelling degree of the block copolymer domains.

Furthermore, there is no necessity to treat the inorganic nanomaterials with intricate surface functionalization techniques that are often time-consuming and can disrupt the self-assembly process due to the presence of organic ligands. The developed protocol can be readily adapted to diverse block copolymer systems and different morphologies, provided that selective swelling of one domain is feasible. Furthermore, it may be extended to accommodate the growth of multiple inorganic nanomaterials *in*-*situ*. Therefore, this work is anticipated to facilitate the proficient fabrication of hybrid photonic structures with unique properties devoted to light-matter interactions and suitable for various technological applications.

## Supporting Information Available

Detailed description of materials, methods, characterization techniques, and supporting data (PDF)
