# Supplementary material for: Crafting Nanostructured Hybrid Block Copolymer–Gold Nanoparticles by Confined Self-Assembly in Evaporative Droplets
Source: ACS Macro Lett. 2024 Sep 23;13(10):1338–44. doi: 10.1021/acsmacrolett.4c00519 (PMC11483756; doi:10.1021/acsmacrolett.4c00519)
Supplement: Supplementary file 1 — mz4c00519_si_001.pdf [file mz4c00519_si_001.pdf]

Supporting Information

Crafting Nanostructured Hybrid Block Copolymer-Gold Nanoparticles by Confined Self-Assembly in Evaporative Droplets

Andrea Escher,[1] Gianluca Bravetti,[2] Simone Bertucci,[1,3] Davide Comoretto,[1] Christoph Weder,[2,4] Ullrich Steiner,[2,4] Paola Lova,[1] Andrea Dodero[1,2,4,]*

[1]Department of Chemistry and Industrial Chemistry, University of Genoa, Via Dodecaneso 31, 16146, Genoa, Italy

[2]Adolphe Merkle Institute, University of Fribourg, Chemin des Verdiers 4, 1700, Fribourg, Switzerland

[3]Photonic Nanomaterials, Istituto Italiano di Tecnologia, Via Morego, 30, 16163 Genoa, Italy

[4]National Center of Competence in Research Bio-Inspired Materials, Chemin des Verdiers 4, 1700, Fribourg, Switzerland

* corresponding author: Andrea Dodero, andrea.dodero@unifr.ch

Materials and Methods

Materials

Poly(styrene)-*b*-poly(2-vinylpyridine) (PS-P2VP) with an average number-average molecular weight (M_n) of 185-*b*-195 kg/mol and a dispersity (Đ) of 1.05 was procured from Polymer Source. The following materials were obtained from Sigma Aldrich: gold(III) chloride trihydrate, sodium borohydride ($NaBH_4$), ethanol, poly(vinyl alcohol) (PVA) with a weight-average molecular weight M_w = 13-23 kg/mol and a hydrolyzation degree of 87-89%, activated basic aluminum oxide, iodine, and chloroform containing 100-200 ppm amylene as a stabilizer. Milli-Q water with a conductivity of 0.055 μS/cm was produced *in situ* and utilized in all experimental procedures.

Methods

Solution preparation

PS-P2VP was dissolved in chloroform, previously treated with aluminum oxide, at a concentration of 10 mg/mL. The solution was magnetically stirred overnight in a sealed glass vial to ensure complete solubilization. Gold(III) chloride trihydrate was dissolved in water or ethanol at a concentration of 5 mM. Sodium borohydride was solubilized in ethanol at a concentration of 5 mM concentration. Polyvinyl alcohol (PVA) was solubilized in Milli-Q water at a concentration of 10 mg/mL or 3 mg/mL by maintaining the mixture at a temperature of 85 ˚C for two hours under stirring.

Particle fabrication

Photonic particles were fabricated as previously described.[1] In detail, 0.25 mL of the block copolymer solution in chloroform was mixed with 2.5 mL of PVA aqueous solution with a 10 mg /mL concentration in a 7 mL screw cap glass vial. The mixture was emulsified using a vortex mixer at 2000 rpm for 15 seconds. The resulting emulsion was rapidly poured into glass Petri dishes (ø = 5 cm) containing 15 mL of PVA aqueous solution with a 3 mg/mL concentration. The Petri dishes were covered to reduce the evaporation rate of the chloroform. After 48 hours, the particles were collected, centrifuged at 10000 rpm for 30 minutes, and washed three times with Milli-Q to remove the PVA surfactant. Subsequently, the particles were redispersed in 2.5 mL of Milli-Q water, resulting in a final suspension concentration of 1 mg/mL.

Particle infiltration and nanoparticle formation

The photonic particles were impregnated with gold(III) chloride by preparing infiltration solutions with varying water-to-ethanol volume ratios (i.e., 100-0, 90-10, 85-15, and 50-50). A fixed volume of the gold precursor solution was added to 0.5 mL of the particle aqueous suspension, and the solvent ratio was then adjusted by adding pure water or ethanol. The particle concentration was maintained at 0.5 mg/mL, and the gold ion concentration was fixed at 1 mM. The resulting mixtures were then subjected to continuous shaking for seven days, during which the gold salt diffused into the photonic particles. The infiltrated particles were centrifuged, washed three times with Milli-Q to remove the excess of gold ions, and redispersed in Milli-Q water at a 0.5 mg/mL concentration. Subsequently, 500 uL of freshly prepared sodium borohydride solution (5 mM in ethanol) were added, and the mixtures were heated to T = 50 °C overnight to induce the formation

of gold nanoparticles. Finally, the particles were centrifuged, washed three times with Milli-Q, and redispersed in Milli-Q water at a 0.5 mg/mL concentration before characterization.

Characterization techniques

Optical microscopy was conducted on the fabricated hybrid particles using a customized microscope (ZEISS Axio Scope.A1) with a CCD camera (Point Grey GS3-U3-28S5C-C) calibrated against a standard white diffuser. A halogen lamp (Zeiss HAL100) was employed as the light source. Micrographs were captured in reflection mode in a bright-field configuration using a 50× objective (Zeiss LD EC Epiplan-Neofluar, NA = 0.8). The reflection spectra of the microparticles were obtained through micro-spectroscopy, which involved coupling the microscope to a diode-array spectrometer (Ocean Optics QEPro) using an optical fiber positioned in confocal alignment with the image plane of the microscope (Avantes QP230-2-XSR, 230 μm core size). An aluminum mirror (Thorlabs PF10-03-G01) was employed as a reference. The photonic microparticles were characterized in aqueous suspensions on optical glass slides covered with a glass coverslip.

The internal morphology of the fabricated hybrid particles was investigated via focused-ion beam scanning electron microscopy (FIB-SEM) using a Thermo Scientific Scios 2 DualBeam FIB-SEM (FEI, Eindhoven, the Netherlands). To ensure optimal conductivity, samples were coated with a 4 nm thick gold layer to ensure good conductivity. The particles were milled using a Ga^+ ion beam with an acceleration voltage of 30 kV and a current of up to 3 nA. The cross-sections were imaged using the integrated SEM Everhart-Thornley (ETD, secondary electrons) and in-lens T1 (A+B

composite mode, back-scattered electrons) detectors, set to a voltage of 5 kV and a current of 0.4 nA. Staining with an iodine aqueous solution was employed for the pristine particles to enhance the contrast between the BCP domains.

Small-angle X-ray scattering (SAXS) and ultra-small-angle X-ray scattering (USAXS) measurements were conducted on the ID02 beamline at the European Synchrotron Radiation Facility (ESRF) in Grenoble.[2,3] Circular samples with an average thickness of 1 mm and a diameter of 4 mm were prepared by drop-casting particle suspensions in polytetrafluoroethylene (PTFE) disks closed on both sides by Kapton tape (DuPont).

Thermogravimetric analysis (TGA) was conducted on the particles using a Mettler-Toledo TGA/DSC 1 STARe System. The degradation profiles were investigated under an 80 mL/min airflow in the 30 – 600 ˚C temperature range with a heating rate of 10 ˚C/min.

Supporting Figures

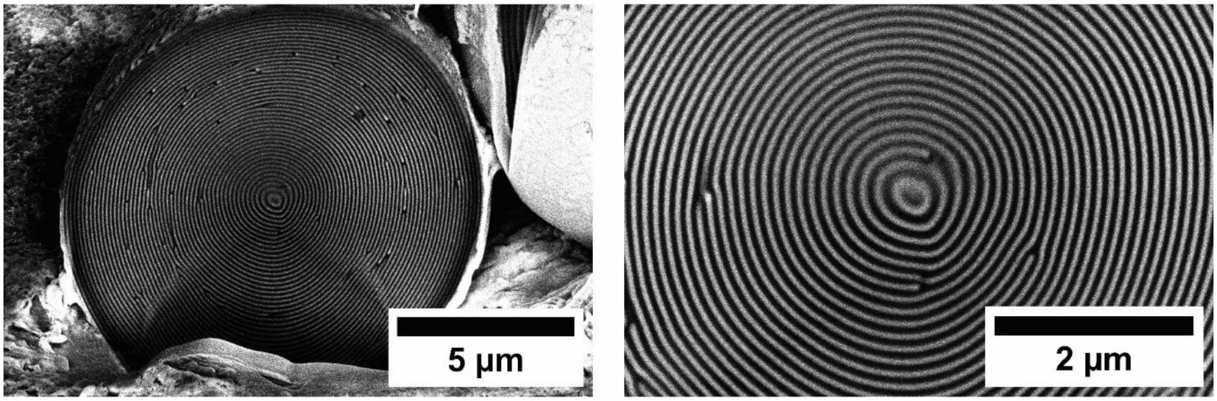

Figure S1. FIB-SEM cross-section micrographs of pristine PS-P2VP after staining with iodine ions. A distinct concentric lamellar structure is evident, exhibiting minimal structural imperfections. The bright layers are identified as P2VP, while the dark ones are attributed to PS. The lamellar thicknesses were d_{PS} = 65 nm and d_{P2VP} = 63 nm.

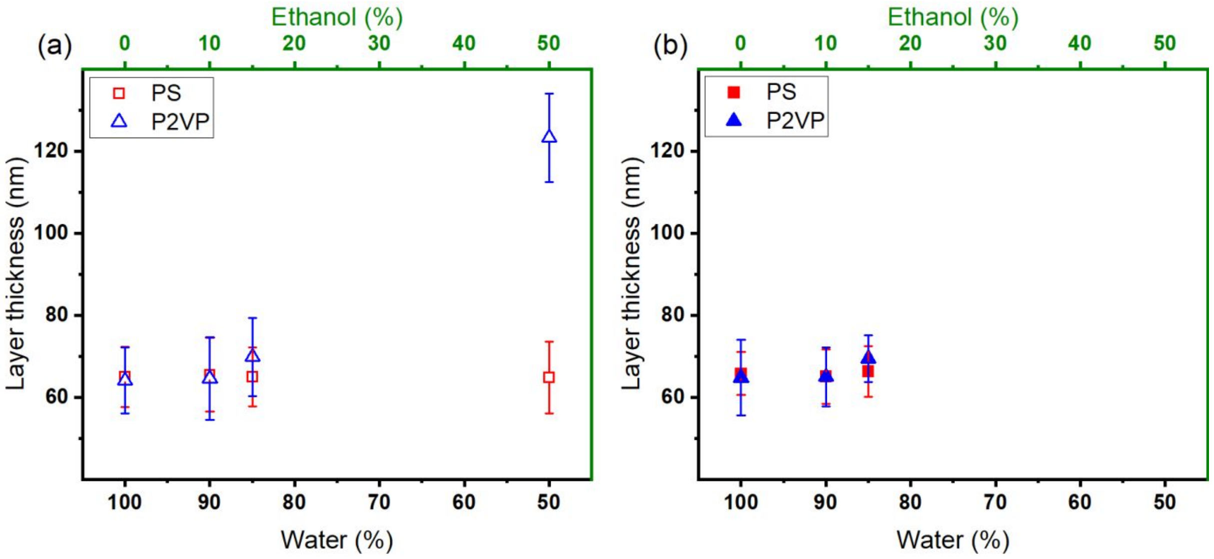

Figure S2. Variation of the layer thickness upon gold ion infiltration in different water/ethanol mixtures before (a) and (b) after chemical reduction with $NaBH_4$. While PS layers do not present significant changes, P2VP layers shows a progressive increased thickness with increasing the ethanol percentage in the infiltration solution. Notably, no significant changes are observed after the formation of the nanoparticles suggesting that the lamellar structure disruption is mostly related to the ethanol-induced swelling.

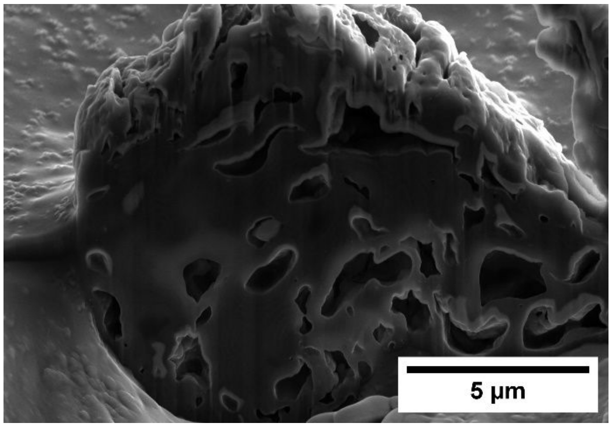

Figure S3. FIB-SEM micrograph of a BCP particle swelled in a 50-50 water/ethanol mixture without gold ions. The formation of several defects is clearly observed, indicating that the uncontrolled swelling of P2VP domains is the primary factor leading to the disruption of the ordered concentric lamellar structure.

Supporting Tables

Table S1. Measured scattering vectors *q* via SAXS and USAXS experiments and calculated domain periodicity D for block copolymer particles infiltrated with gold ions in different water/ethanol mixtures. Increasing the ethanol content in the infiltration solution leads to an increment of the domain periodicity due to the progressively higher swelling of P2VP layers.

Sample (Infiltrated)	q (nm^{-1})	D (nm)
**100-0**	0.043683	143
**90-10**	0.042147	149
**85-15**	0.042002	150
**50-50**	0.033717	186

Table S2. Residual masses at T = 600 ˚C for hybrid BCP particles infiltrated in different water/ethanol mixtures after the chemical reduction of gold(III) ions. Increasing the ethanol content in the infiltration solution leads to a greater content of AuNPs in the final block copolymer structure due to a more efficient swelling of P2VP domains.

Sample (Reduced)	Residual mass at 600 ˚C (%)
100-0	58.7
90-10	60.1
85-15	67.8
50-50	71.6

REFERENCES

(1) Dodero, A.; Djeghdi, K.; Bauernfeind, V.; Airoldi, M.; Wilts, B. D.; Weder, C.; Steiner, U.; Gunkel, I. Robust Full-Spectral Color Tuning of Photonic Colloids. *Small* **2023**, *19* (6), 2205438. https://doi.org/10.1002/smll.202205438.

(2) Bauernfeind, V.; Bravetti, G.; Dodero, A.; Vogler-Neuling, V. V. Bio-Inspired Photonic Structures for Optical Materials [Dataset]. *Eur. Synchrotron Radiat. Facil.* **2026**. https://doi.org/10.15151/ESRF-ES-1330737234.

(3) Narayanan, T.; Sztucki, M.; Zinn, T.; Kieffer, J.; Homs-Puron, A.; Gorini, J.; Van Vaerenbergh, P.; Boesecke, P. Performance of the Time-Resolved Ultra-Small-Angle X-Ray Scattering Beamline with the Extremely Brilliant Source. *J. Appl. Crystallogr.* **2022**, *55* (1), 98–111. https://doi.org/10.1107/S1600576721012693.
